# Study of the Simultaneous Utilization of Mechanical Water Foaming and Zeolites and Their Effects on the Properties of Warm Mix Asphalt Concrete

**DOI:** 10.3390/ma13020357

**Published:** 2020-01-12

**Authors:** Anna Chomicz-Kowalska, Krzysztof Maciejewski, Mateusz M. Iwański

**Affiliations:** 1 Department of Transportation Engineering, Faculty of Civil Engineering and Architecture, Kielce University of Technology, Al. Tysiąclecia Państwa Polskiego 7, 25-314 Kielce, Poland; kmaciejewski@tu.kielce.pl; 2 Department of Building Engineering Technologies and Organization, Faculty of Civil Engineering and Architecture, Kielce University of Technology, Al. Tysiąclecia Państwa Polskiego 7, 25-314 Kielce, Poland; matiwanski@tu.kielce.pl

**Keywords:** WMA, asphalt concrete, natural zeolite, foamed bitumen, moisture susceptibility

## Abstract

The paper aimed at assessing the feasibility of using natural zeolites as a mineral filler substitute for asphalt mixtures produced at around 120 °C temperatures with a water foamed binder and compacted at 100 °C. The tests utilized the AC 16 asphalt concrete mixture intended for the binder and base course with the mineral filler fraction amounting to 4% by wt. comprising limestone dust and zeolites (when added). A reference hot mix and warm mix with foamed bitumen were compared to two mixes with zeolites, with one containing 0.4% of a water-modified (20% moisture content) zeolite and the second containing 1.0% of natural air-dried zeolite. The investigations included: assessment of campactability using a gyratory compactor, air void content, indirect tensile strength before and after conditioning with one freeze-thaw cycle, and the resulting resistance to moisture and frost damage. The mixtures with zeolites exhibited decreased compactability when compared to reference mixes, which the Marshall samples confirmed. The mechanical properties have also deteriorated in zeolite-bearing mixtures, which was partially accounted to the decreased compaction level. It was concluded that the temperature of the mixture production was too low for the zeolite water to significantly improve the compactablity of the asphalt mixture and its mechanical parameters.

## 1. Introduction

The production of traditional hot mix asphalts (HMA) is very energy consuming, mainly because of the high production temperatures, which typically range from 160 to 190 °C. The actual production temperature depends chiefly on the type of used asphalt and its viscosity at high temperatures, which is decisive in terms of coating the mix’s components. As such, paving with asphalt mixtures is a source of emission of greenhouse gases and other substances with a negative impact on the environment. Human labor conditions at HMA paving over long periods of time are also a concern. Due to the above, the production of HMA’s is not considered to be indifferent to the environment, but these effects can be reduced with the use of adequate technical measures. Therefore, actions are taken to change the asphalt production technologies to make them more environmentally friendly and contributing to worker safety. The effect of these activities is the development of new technologies of producing warm mix asphalts (WMA) and research on the half-warm mix asphalts (HWMA) [1,2,3,4,5].

Currently, the greatest technological developments are seen in the field of warm mix asphalt, in which mainly Fischer-Tropsch synthetic waxes or Montan-type waxes and chemical state-of-the-art warm mix additives are used. The use of a water foamed binder is another popular method of achieving this goal. In this scope, it is possible to distinguish two main technology groups: foaming using water injected directly into the binder (mechanical foaming) and by the use of zeolites or other similarly acting granular materials (e.g., mesoporous silica [6]) added to mineral-bitumen mixes as a mean for introducing foaming water.

The bitumen foaming technique for producing bitumen-aggregate mixtures was at first used for producing road paving mixes at ambient temperatures with low-quality aggregates and recycled road base material, but now it is also used in WMA and HWMA techniques [7]. The investigations of bitumen water-foaming for production of WMA came in the late 1990’s and early 2000’s with the emergence of other warm mix techniques [8]. The sudden expansion of water contacting hot bitumen results in the formation of bitumen foam with greatly increased volume and surface area, decreased viscosity, and enhanced coating abilities. These effects in conjunction with adequate aggregate temperature permit the production of high-quality asphalt mixtures at lowered temperatures [4,5,9,10,11].

Zeolites are crystalline, hydrated aluminosilicates of alkali elements (Na, K, less often Li), alkali soils (Ca, Mg, Ba, more rarely Sr), or other monovalent or polyvalent metals. These materials are micro-porous solids with exchangeable cations and zeolite water in the crystalline network channels. The zeolite voids are sufficiently big to enable diffusory permeation of not only single atoms, but whole chemical compound particles without changing the volume of the crystal structure, which makes zeolites a medium for certain chemical compounds and it enables their use, e.g., for absorbing unwanted chemical reaction products [12,13,14]. Zeolites possess unique absorptive properties, which are mainly related to their porous structure that allows for water release and absorption. Water desorption is a function of time and temperature. Thanks to their properties, zeolites became widely used in the global economy, not only as WMA additives, but also, among others, in the cement concrete technology [15,16,17], as sorbents in the environmental protection and household chemical products technologies [18,19,20,21] and as catalysts in the chemical and oil industry [22].

The naturally occuring zeolites include several dozen various mineral variants, among which the most common and used are: clinoptilolite, modenite, analcime, or chabasite. Many other variants are only known from singular aggregations [23]. Due to the high demand for zeolites, they are also produced on an industrial scale through synthesis. Thus far, over 400 zeolites were obtained this way [24].

A specific feature of zeolites is the occurrence of the so-called “zeolite water”, which is continuously released into the environment, along with their heating up to approx. 400 °C. This fully reversible process, does not cause changes in the volume of zeolite crystal structure. The potential occurrence of this phenomenon was observed in terms of the possibility of improving the workability of asphalt mixtures. Zeolites are currently a popular WMA additive, which are usually used as a replacement for a fraction of the filler aggregates. The technology of producing asphalt mixes with zeolites does not differ from traditional technologies [25], which is why its implementation is possible without incurring substantial financial expenses. The mixing temperature causes the gradual release and vaporisation of the zeolite’s pore water, thereby causing asphalt foaming. At the same time, zeolites do not change the properties of the bitumen binder.

The research that was conducted by Lai et al. [26] showed that the the *Advera* synthetic zeolite gradually releases water over time, whereas the stabilisation of this process takes place after 20–40 min. More zeolite water is released along with increasing temperature. The improvement of the asphalt mixture’s workability takes place after a minimum of 20 min. from the indroduction of zeolites to the mixture. The optimal temperature that is required for effective asphalt foaming amounts to 110–120 °C [26].

The analysis of the results presented in A. Woszuk’s paper [27] on compactability testing in the gyratory compactor indicates that the optimal content of the NaP1 synthetic zeolite amounted to 0.5% by wt. of mineral mix and 0.4% in the case of water modified zeolite. The optimal quantity was 1.0% by wt. of mineral mix and 0.4% for water-soaked mineral in relation to the weight of the mineral mix when using the clinoptilolite natural zeolite.

Analysis of the possibility of simultaneous use of bitumen mechanical foaming (using water injection) and zeolites were not found in the literature, therefore the purpose of this paper was to evaluate the feasibility of the simultaneous use of the mechanical water-foaming and the utilization of zeolites in the production of WMA asphalt concrete mixtures at ca. 120 °C. The investigations included an assessment of the compactability and basic mechanical properties (indirect tensile strength, resistance to moisture, and frost damage) of an AC 16 mix designed to withstand a medium traffic load (up to 7.3 × 10^6^ 100 kN equivalent single axle loads in the design period).

## 2. Aims and Design of Experiment

### 2.1. Purpose and Scope of Research

The research subject was to test the feasibility and effects of using the natural zeolite additive for asphalt mixtures produced and then compacted in lowered temperatures along with the mechanical bitumen water foaming method. The main purpose of laboratory testing was to determine the impact of the zeolite additive on selected physical and mechanical properties of the WMA and water foamed asphalt mixtures as well as on its compaction.

The utilization of natural zeolite was evaluated while using the AC 16 asphalt concrete mix based on limestone and quarzite aggregates, with the use of the reference mixes that are produced in the hot mix asphalt technology *HMA_Ref_* and warm-mix method with foamed bitumen only *WMA_Ref_*. The only differences concerned the production temperatures (*HMA_Ref_*: ca. 160 °C, *WMA* mixes: ca. 120 °C), compaction temperatures, dosed binder form (liquid in the *HMA_Ref_* mix and water foamed in the WMA mixes), and additives in the composition of the asphalt concrete. Table 1 presents the materials used in the formulation of the asphalt concrete mixes. In the case of zeolite dosage, the mixture composition was corrected by lowering the mineral filler content in the mineral mix by the quantity of the incorporated zeolite additive. The laboratory testing utilised zeolite in two forms: dry (unmodified) and modified with water (up to 20% moisture content).

The analysis covered the following mixes:

*HMA_Ref_* - reference Mix Produced as Hot Mix Asphalt Using the 35/50 Asphalt with 0.3% (by wt.) of Adhesion Promoter,
*WMA_Ref_* - reference mix produced at lowered temperature with water foamed asphalt produced from 35/50 binder, with 0.6% (by wt.) of adhesion promoter,
*WMA_nz,0.4%S_* - mix produced at lowered temperature with water foamed asphalt produced from 35/50 binder, with 0.6% (by wt.) of adhesion promoter, including natural zeolite in the amount of 0.4% of the mix weight, soaked to 20% moisture content,
*WMA_nz,1.0%D_* - 35/50 binder, with 0.6% (by wt.) of adhesion promoter, including natural zeolite in the amount of 1.0% of the mix weight and 7.4% moisture content.

As specified before, it was decided to evaluate the impact of dosing of natural zeolite in two variants: dry (at its natural moisture content in air-dry conditions, *D*—dry) and soaked in water (up to 20% moisture content by weight, *S*—saturated). The quantity of water soaked zeolite amounted to 0.4% (by wt.) of the mineral mix, whereas the dry zeolite was used in the quantity of 1.0% (by wt.). These values were chosen based on the analysis of technical literature [27]. The degree of saturation was chosen in this way, so that the total quantity of water delivered to the asphalt mixture by means of zeolite was in both cases comparable. Table 2 presents adequate calculations.

It was first necessary to determine the impact of using the adhesion promoter on the properties of the binder and asphalt foam due to the use of various contents of the adhesion promoter and foamed bitumen binder in the analysed mixes. The testing of asphalt concrete was conducted during the second stage of works.

### 2.2. Evaluation of the Properties of Bituminous Binders

The purpose of the first stage was to determine the impact of the Wetfix BE [28,29] adhesion promoter (referred to as WBE) on the basic properties of the 35/50 penetration paving bitumen prior to foaming, the impact of this additive on the foaming process, and to determine the optimal foaming water content for this binder. The investigated 35/50 binder is commonly used in central and eastern Europe for producing asphalt concrete mixtures that are intended for road pavements with medium traffic (0.5 × 10 ^6^ ≤ ESAL_100 kN_ ≤ 7.3 × 10 ^6^).

The liquid adhesion promoter was dosed in the following quantities: 0.3%, 0.6%, and 0.9% (by wt.). The quantitative range of using the surfactant additive was formulated based on the high silicon dioxide content (SiO_2_ > 60%) in the aggregate and on the lowered technological temperatures used in WMA mixes with foamed asphalt. Increased amounts of surface active chemical agents are also recommended due to the necessity of ensuring the asphalt pavement’s water and frost resistance, exploitational durability, and ensuring adequate foaming performance.

The 35/50 road paving bitumen was subjected to the determination of the effects of WBE adhesion promoter on the basic parameters describing its consistency, thermal sensitivity and low temperature performance based on the following laboratory testing:

- penetration at 25 °C (*Pen_25_*) acc. to EN 1426:2015-08 [30], 10 replicates
- softening point acc. to the “Ring and Ball” method (*T_R&B_*) acc. to EN 1427:2015-08 [31], four replicates
- Fraass breaking point (*T_Fraass_*) acc. to EN 12593:2015-08 [32], three replicates.

Based on the obtained results, the following parameters were determined by using analytical methods:

- penetration index *PI* based on EN 12591:2010 [33]
- temperature plasticity range (*PR*) acc. to EN 14023:2011 [34]

All of the binders were also subject to measurements of the following asphalt foam’s physical parameters in accordance to procedures presented in [1,35,36,37,38,39]

- *ERm*—maximum expansion ratios
- *HL*—half-life.

The bitumen foam parameters were measured at five different foaming water contents (FWC) ranging from 1.5% to 3.5%. The measurements were conducted four times for each of the FWC’s. The adhesion promoter used in the testing was mixed with the binder directly in the Wirtgen WLB 10S device prior to the foaming process, which ensures the binder’s homogenisation. The foaming conditions (air and water pressure, bitumen temperature) were in accordance with [38,40,41]. All of the test samples were prepared according to EN 12594:2014-12 [42].

Based on the aforementioned comprehensive test results and author’s prior experiences, the WBE additive content was determined for producing HMA and WMA mixes with foamed asphalt.

### 2.3. Evaluation Of The Properties of Foamed Warm Mix Asphalt with Zeolites

All of the experiments utilized the same AC 16 asphalt concrete mix designed with a maximum aggregate size of 16 mm intended for binding and base courses of pavements with medium design traffic. The mix was designed in accordance to EN 13108-1:2008 [43] and Polish technical requirements WT-2 [44].

The 35/50 penetration paving bitumen was used in all of the asphalt mixtures. It was necessary to use an adhesion promoter (WBE) due to the fact that the designed mineral mix composition featured quartzite aggregates, where content in relation to the binder’s weight amounted to 0.3% in the *HMA_Ref_* reference mix and 0.6% in the WMA mixes in relation to the binder’s weight, which was determined based on previous experiences [45,46] and the test results included in the latter part of the paper.

The scope of the laboratory works included testing of the following physical and mechanical properties of the AC 16 asphalt concrete mixes:

- density *ρ_mv_* acc. to EN 12697-5:2010 [47],
- bulk density *ρ_bssd_* acc. to EN 12697-6:2012 [48],
- air void content *V_a_* acc. to EN 12697-8:2005 [49],
- indirect tensile strength *ITS_dry_* in air-dried conditions, indirect tensile strength *ITS_freeze-thaw_* after conditioning with a single freeze-thaw cycle, and
- water and frost resistance *ITSR* acc. to EN 12697-12:2008 [50] and acc. to WT-2 (Appendix no. 1) [44]—the procedure was specified in detail in paper [4] (procedure B, Section 2.3).

In addition, the compaction performance of the asphalt mixtures was analysed while using a gyratory compactor (Controls S.p.A., Milan, Italy) acc. to EN 12697-31 [51].

## 3. Materials

### 3.1. Natural Aggregates

Natural aggregates were chosen based on EN 13043 [43] and domestic requirements (WT-1 [52], WT-2 [44]), as intended for the binding layer and the subbase of pavement with a medium traffic load, in order to develop the reference composition of the AC 16 asphalt concrete mix.

**Figure 1:**
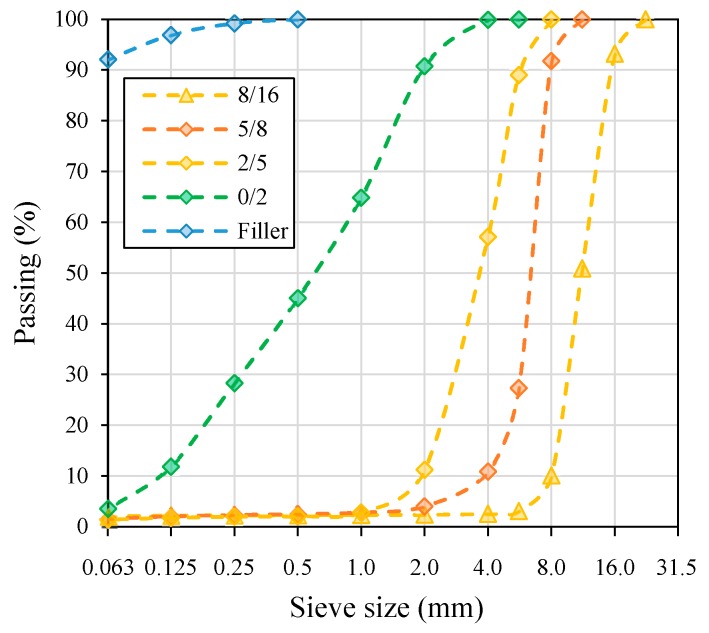
Grading curves of the mineral aggregates used in asphalt concrete (AC 16) mixtures.

Table 3 and Figure 1 present the results of air-jet sieving of the filler aggregate in acc. to EN 933-10 [53] and the remaining aggregate gradation determined by the sieving method acc. to EN 933-1 [54].

### 3.2. Bitumen Binder and Surfactant

All of the asphalt concrete mixes utilised the 35/50 road paving bitumen, which, in accordance with the requirements of WT-2 [44], is recommended for the binding layer and subbase of pavement with a medium traffic load. As mentioned, the AC 16 mix utilised acidic aggregates (quartzites), it was therefore necessary to use a surface active agent (adhesion promoter) to improve the aggregate’s wettability with the bitumen binder and, thus, provide adequate bitumen-aggregate adhesion. The WBE adhesion promoter was used for this purpose and its content in relation to the bitumen weight amounted to 0.3% for the reference hot mix asphalt (*HMA_Ref_*) and 0.6% for the warm mix asphalts.

### 3.3. Zeolites

**Figure 2:**
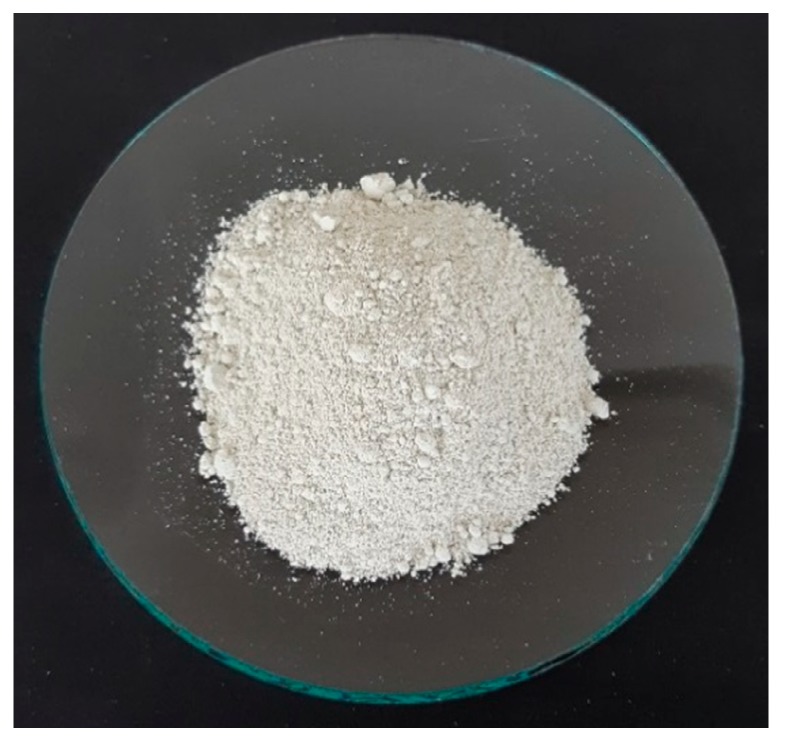
Macroscopic image of the ZeoBau 0–50 μm natural zeolite.

**Figure 3:**
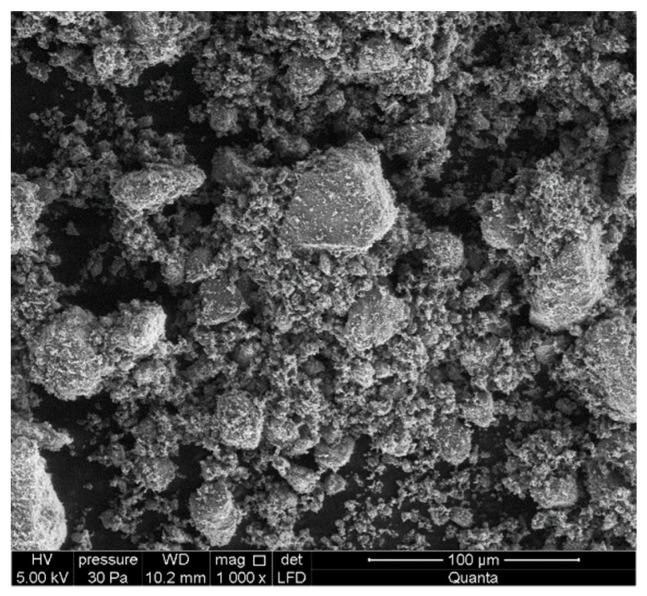
Scanning electron microscope image of the natural zeolite (Quanta FEG 250 SEM).

**Figure 4:**
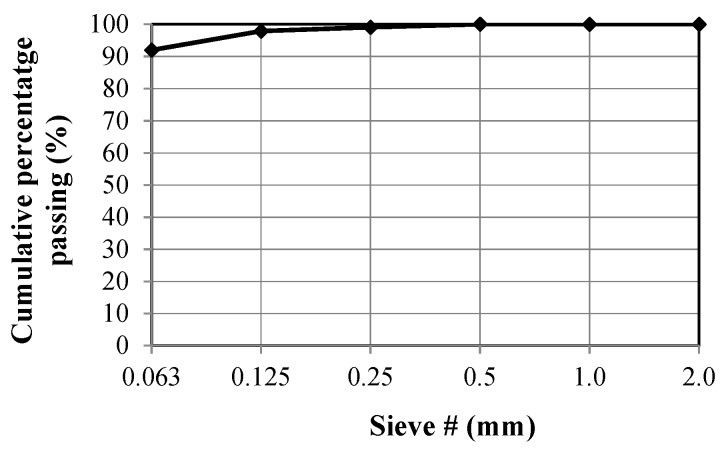
Grading curve of the ZeoBau 0–50 μm natural zeolite.

The ZeoBau natural zeolite (0–50 μm) was utilized in the laboratory tests. After milling to adequate granulation and drying to remove excess water, this mineral presents a form of grey powder, as presented in Figure 2. Figure 3 presents its microscopic image with grains mostly not exceeding 50 μm, which corresponds to the results of the granulometric analysis conducted while using the air-jet sieving method acc. to EN 933-10 [53], as presented in Figure 4.

**Figure 5:**
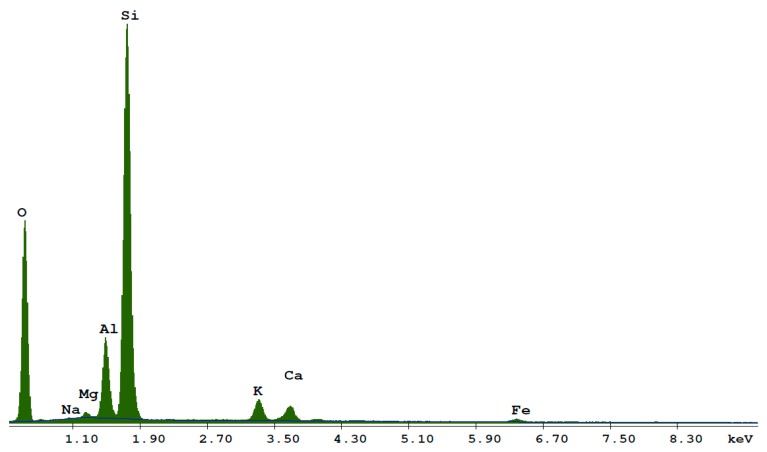
The EDX spectrum of the investigated zeolite sample.

Figure 5 and Table 4 present the results of the natural zeolite’s EDX (energy dispersive X-ray spectroscopy) analysis using the standardless ZAF quantification method in the Quanta FEG 250 Scanning Electron Microscope (FEI / Thermo Fisher, Hillsboro, OR, USA). The dominant elements of natural zeolite are O, Si, and Al, which correspond to the chemical composition specified by the manufacturer. The analysed sample demonstrated additional presence of potassium, calcium, iron, and trace amounts of magnesium and sodium.

### 3.4. Asphalt Concrete Mix

As mentioned before, the investigated asphalt concrete mixes were designed with identical compositions, with changes only in the amounts of the filler and zeolites added. Table 5 provides the framework composition of all the mineral mixtures (mm) and asphalt mixtures (am).

**Figure 6:**
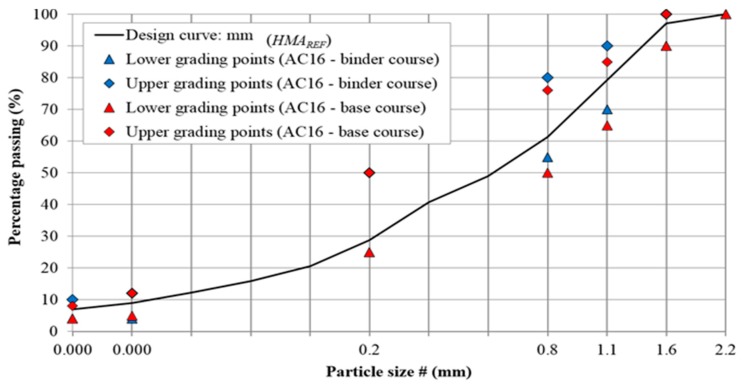
Grading curve of the AC 16 mineral mix with boundary points in accordance to WT-2 [44].

Table 6 presents the digest of sieving analysis, i.e., filler, (<0.063 mm), sand (from 0.063 to 2 mm), and coarse fraction (>2 mm) content, as well as the density of the designed mineral asphalt mixtures. Figure 6 represents the final grading of the mineral mix along with the boundary grading points.

The aforementioned results indicate that the presence of zeolite in the mineral mixes did not cause changes in their granulometry in comparison to reference mixes and only the mineral mixes’ density (ρ_a_) has slightly changed.

All of the utilised mineral materials met the requirements stated in relevant technical documents [44,52] concerning their use in pavements’ binding and base courses.

The design and production of asphalt mixtures featured the use of recommendations specified in relevant technical documents [55]. Prior to the production of asphalt mixtures, the coarse and fine aggregates were heated for 4 h at a temperature of 170 °C (HMA) or 130 °C (WMAs). The adhesion promoter was added to the asphalt before producing the asphalt mixture and its uniform mixing with the binder was ensured. Zeolites were added in adequate proportions to the filler aggregate and then mechanically mixed for 2 h. This “mixed filler” was added to the mix without pre-heating.

The following technological temperatures were used during the production and compaction of asphalt mixtures:

- reference hot-mix *HMA_Ref_* (road paving butimen: 35/50 + 0.3% WBE):
  - bitumen temperature 35/50: 170 °C,
  - temperature of produced mineral-bitumen mix: 160 °C–170 °C,
  - specimen compaction temperature: 140 °C–145 °C,
- reference warm-mix *WMA_Ref_* (road paving butimen: 35/50 + 0.6%WBE):
  - bitumen temperature prior to foaming: 155 °C,
  - temperature of produced mineral-bitumen mix: 120 °C–125 °C,
  - specimen compaction temperature: ca. 100 °C.
- warm mixes *WMA_Ref_*, *WMA_nz_*_,0.4%*S*_ and *WMA_nz_*_,1.0%*D*_ (road paving butimen: 35/50 + 0.6%WBE):
  - bitumen temperature prior to foaming: 155 °C,
  - temperature of produced mineral-bitumen mix: 120 °C–125 °C,
  - specimen compaction temperature: ca. 100 °C.

## 4. Results and Discussions

### 4.1. Bituminous Binder Properties Prior to Foaming

Table 7 and Table 8 present the basic properties of the 35/50 penetration binder with the addition of the WBE adhesion promoter in concentrations from 0.0% to 0.9%.

The use of the WBE additive caused an increase in penetration from 36.6 (0.1 mm) at the WBE’s concentration of 0.0% to 42.9 (0.1 mm) at the WBE’s concentration of 0.9% in the bitumen binder. In the case of the softening and breaking points, a slight impact of using the WBE additive at 0.3% and 0.6% to the 35/50 binder was noted. The difference in results for the first feature did not exceed 1 °C, whereas it amounted to 1.4 °C for *T_Fraass_*.

When evaluating the calculated penetration indexes that enable initial asphalt classification in terms of their temperature sensitivity, it was observed that these values increased as a result of adding the surface active agent. These amounted to −0.9 for pure asphalt, whereas the bitumen with the adhesion promoter in concentrations of 0.3%, 0.6%, and 0.9% obtained a PI values of −0.7.

In road construction, the usually used of bitumens range in *PI* from -2.0 to +2.0, but it is recommended to use the asphalts with the *PI* from −1.0 to +1.0 [56]. Based on the obtained values of this parameter, the tested binders can be classified as rheological sol-gel with the penetration index from −2 to +2. The introduction of the adhesion promoter resulted in a slight decrease of the temperature plasticity range of the base road paving bitumen. The *PR* value was decreased from the initial 68.0 °C to 65.7 °C at the 0.9% concentration of the additive.

It can be observed that the highest coefficient of variance was recorded for *T_Fraass_* (11.2%) and the lowest for *T_R&B_* (average value of 0.32%) when analysing the descriptive statistics. On the other hand, the *Pen_25_* parameter had an average value of 3.41%.

An analysis of variance was carried out in the statistical evaluation of the impact of the WBE additive content on the basic parameters of bitumen binders, the results of which are presented in Table 9.

It is possible to state that the WBE adhesion promoter had a statistically significant impact on the *Pen_25_* and *T_R&B_* parameters, because the *p*-value < 0.05 was achieved, after the analysis of the obtained *p*-values for the *F* statistic (Table 9), with the assumed significance level of α = 0.05. Statistically significant impact of the WBE was not proven only in the case of the last feature (*T_Fraass_*).

### 4.2. Foamed Bitumen Binder Properties

Table 10 presents the characteristics of the foamed reference 35/50 penetration grade road paving bitumen. The analysed binder was characterised by the lowest expansion ratio at low foaming water contents, which increased along with the FWC increase. In the case of the bitumen foam half-life, this dependency was inverted, and the HL value proportionally decreased to the increase in the foamed water content. At the the lowest FWC = 1.5% the recorded bitumen foam characteristics amounted to *ERm* = 7.3 and *HL* = 38.3 s, while, at the highest FWC = 3.5%, the expansion ratio was more than doubled (16.3) and half-life was almost halved (20.3 s).

**Figure 7:**
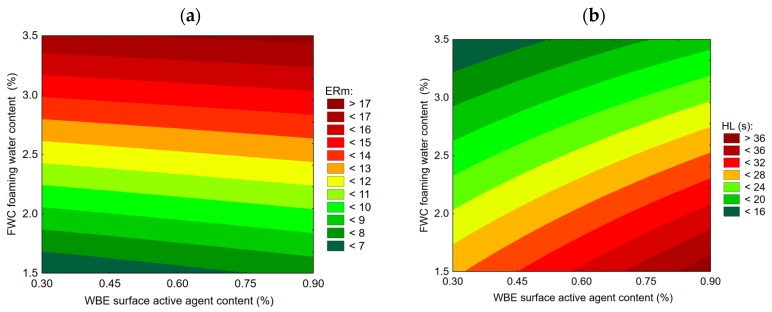
The foaming performance of 35/50 penetration bitumen as a function of foaming water and WBE contents: expansion ratio (**a**), half-life (**b**).

To evaluate the impact of the WBE adhesion promoter on the foaming properties of the 35/50 bituminous binder, its foaming performance was evaluated at different levels of foaming water content and WBE content while using response surface method with first order and interaction terms. Figure 7a,b present the response surfaces derived for *ERm* maximum expansion and the *HL* half-life as a function of foaming water content and the WBE additive content. Table 11 and Table 12 present the statistical evaluation of these models’ parameters.

The results of foaming of the 35/50 asphalt with the WBE adhesion promoter additive that are presented in Figure 7a indicate a very limited impact of the adhesion promoter content on the maximum expansion, which is, therefore, mainly affected by the foaming water content (Table 11). In the case of the bitumen foam half-life, it was, however, demonstrated that increasing the WBE content caused a significant increase in the foam’s half-life (Figure 7b, Table 12). It can be concluded that adding WBE agent resulted in minor decrease of maximum expansion and a significant increase in half-life.

When comparing the results of the reference binder and the 35/50 bitumen with the initial addition of 0.3% of WBE, the adhesion promoter had minor impact on expansion and it significantly shortened the bitumen foam half-life.

**Figure 8:**
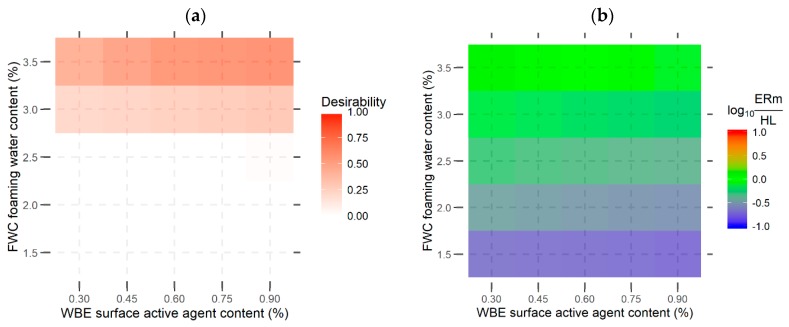
Evaluation of the foaming features of the 35/50 asphalt with the WBE additive: (**a**) desirability function, (**b**) the log_10_ of the ratio of foam’s maximum expansion and half-life.

Figure 8a,b present the results of evaluation and optimisation of the tested binders’ composition in the function of the WBE additive content and the foaming water content, based on their foaming performance. Two independent methods were used to simultaneously evaluate the foaming performance (*ERm* and *HL*)—the desirablity function (Figure 8a) and the method of designating the intersection of the *ERm* and *HL* foaming characteristics (Figure 8b), as specified in publications [40,41,57].

The applied desirability function assigned a certain value of desirability *D* to each experiment point (defined by a combination of the foaming water content and WBE content). The desirability is only assigned a positive non-zero value when all of the specified boundary conditions were simultaneously met (i.e., *ERm* ≥ 12 and *HL* ≥ 10 s). The desired *ERm* and *HL* levels were based on previous experiences and literature [40,41,57,58]. The desirability function applied in this case is assigned with the following values:

- 0: if *ERm* <12 or *HL* < 10 s,
- 0 to 1: if 20 > *ERm* ≥ 12 or 16 s > *HL* ≥ 10 s,
- 1: if *ERm* ≥ 20 s and *HL* ≥ 16 s.

The second method that was employed for evaluating the foaming was based on designating the intersection of the *ERm* and *HL* foaming features (numeric equality of *ERm* and *HL*), and mathematically was performed by calculating the $log_{10}\left(\frac{ERm}{HL}\right)$, which was equal to:

- 0: for *ERm* = *HL* (when *ERm* and *HL* are equal, i.e., they intersect),
- 1: for *ERm* = 10∙*HL*,
- −1: for 10∙*ERm* = *HL*.

The applied method that was based on presenting the common logarithm from the *ERm*’s and *HL*’s ratio on the chart was found to be convenient for representig the ratio of the two features on a plane with the use of a colour scale.

The values of the desirability function *D* that are presented in Figure 8a indicate that meeting the boundary foaming parameters requires the 35/50 binder with the WBE additive to be foamed with at least 3% of foaming water content. The additive content had minor impact on the quality of the asphalt foam evaluated using the desirability function due to the minor impact of the WBE additive content on the maximum expansion (and its relatively low values). The intersection of binder foaming characteristics (*ERm* and *HL*) occurred at ca. FWC = 3.5% and the WBE additive content = 0.30–0.45 (Figure 8b).

When considering the prior experiences and the aforementioned results of bitumen binder testing, further laboratory testing (for WMA asphalts with foamed asphalt) featured the use of a binder with 0.6% of the surfactant content, characterised by *ER* = 14.1, *HL* = 20.5 s at FWC = 3.0%.

### 4.3. AC 16 Mixes’ Compactability Test Results

It was decided to firstly conduct the compactability testing in the gyratory compactor acc. to EN 12697-31 in order to determine the usefullness of the natural zeolite additive for the AC 16 asphalt concrete mix produced with water foamed asphalt and with lowered process temperatures [51].

**Figure 9:**
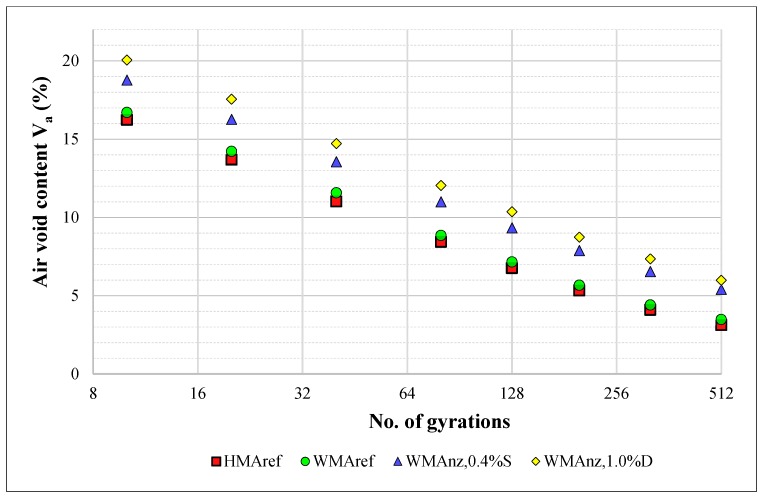
Results of the compactability analysis of the AC 16 mixes compacted in the gyratory compactor; mean values of the samples’ air voids content *V_a_*.

Figure 9 and Table 13 present the compactability of the tested *HMA_ref_* and *WMA_ref_* reference mixes as well as the ones including 0.4% soaked natural zeolites (with 20% moisture content by wt.) and 1% of zeolites in a air-dry state, *WMA_nz,_*_0.4%*S*_ and *WMA_nz_*_,1.0%*D*_, respectively.

When analysing the presented compactability curves, it is possible to state that both reference mixes were characterised by similar compactability. The introduction of zeolites into the mixtures with foamed bitumen had a substantial impact on their compaction process, resulting in an increased air void content during the whole compaction process, on average by approximately 2.1 to 3.1% in the case of soaked and air-dry zeolites, respectively. It was also found that the water-soaked zeolites added in smaller amounts resulted in more efficient compaction when compared to the 1.0% addition of air-dry zeolites.

There are at least two possible explanations for these effects. On one hand, the temperatures of WMA mixes’ production and compaction (approx. 120 °C and approx. 100 °C, respectively) were not high enough to cause zeolite water to release at sufficient rate. Thereby, the additional effects of asphalt foaming due to adding zeolite could be insignificant and, furthermore, the forming asphalt mastic became stiffened due to adding zeolite, which is confirmed by other researchers’ studies [27]. On the other hand, the observed phenomenon could be also related to the reported findings [58] about the negative impact of higher foamed water contents on the asphalt mixture workability—the addition of zeolites has substantially increased the quantity of water delivered to the asphalt mix.

### 4.4. Results of Volumetric and Mechanical Tests Performed on the AC 16 Mixtures

#### 4.4.1. Air Void Content (*V_a_*), Indirect Tensile Strength (*ITS_s_*, *ITS_freeze-thaw_*), Water and Frost Resistance (*ITSR*)

The obtained volumetric and mechanical properties of the investigated asphalt mixtures are presented as bar charts that are supplemented with error bars denoting standard deviations of the respective means.

**Figure 10:**
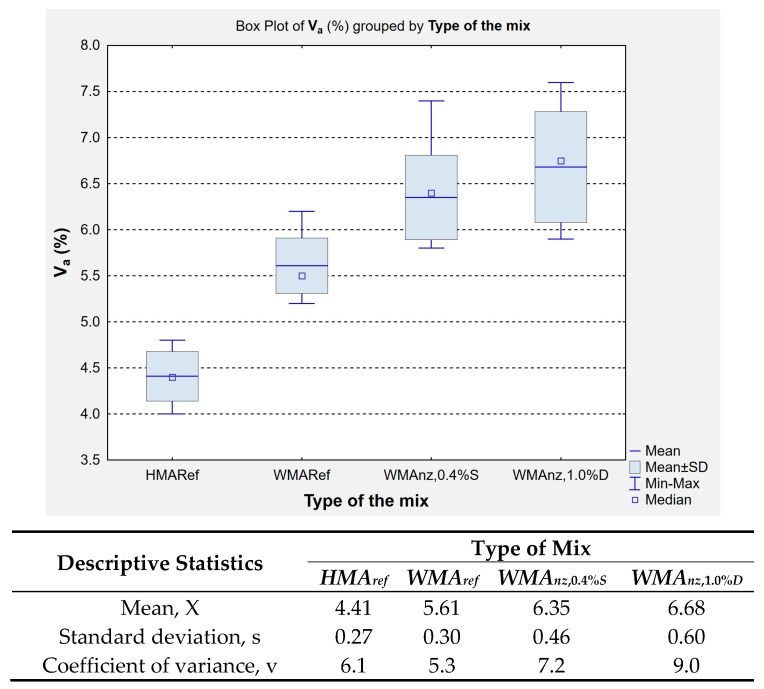
Results of air void content (*V_a_*) determination in Marshall samples produced from the investigated AC 16 mixtures.

Figure 10 presents the results of air void content measured in Marshall compacted samples.

The determination of air void contents *V_a_* in the Marshall compactor (75 impacts per side, 2 × 75 in total) samples confirmed the findings that were obtained in the compactability testing using the gyratory compactor, which indicated substantial impact of the natural zeolite (air-dry and water-soaked) addition to the foamed WMA mixes on their compaction. The zeolite-containing *WMA_nz_*_,0.4%*S*_ and *WMA_nz_*_,1.0%*D*_ mixtures obtained visibly higher air void contents than it was in the case of both reference mixes (*HMA_ref_* and *WMA_ref_*). The introduction of zeolites to the foamed WMA mixtures resulted in increased air void contents of the tested samples with only small difference in *V_a_* over the two mixtures, with *V_a_* = 6.35% and *V_a_* = 6.68% in the *WMA_nz_*_,0.4%*S*_ and *WMA_nz_*_,1.0%*D*_ mixtures, respectively.

**Figure 11:**
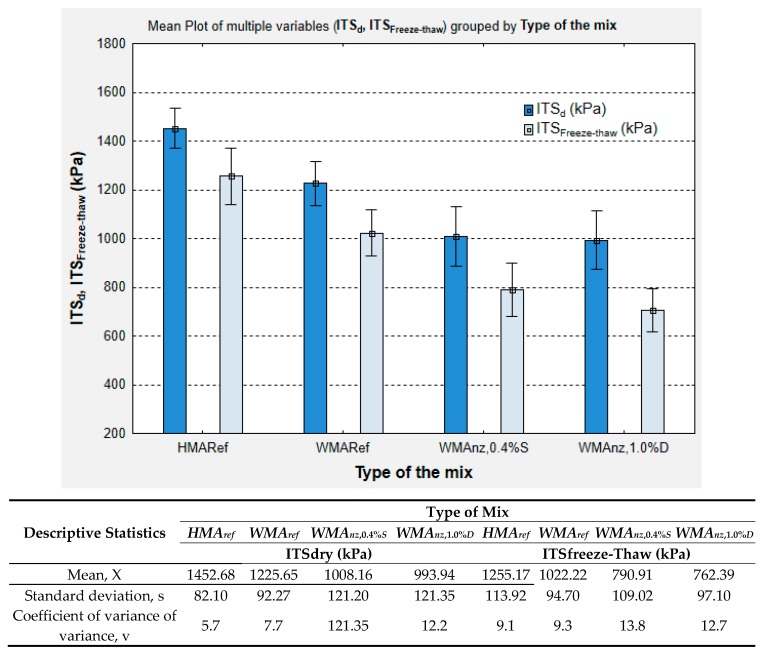
Results of indirect tensile strength (*ITS_dry_* and *ITS_freeze-thaw_*) determination in Marshall samples produced from the investigated AC 16; error bars represent ±1 standard deviation from the mean.

Figure 11 presents the results of indirect tensile strength testing of dry samples and samples subjected to conditioning with a single freeze-thaw cycle, produced from the tested asphalt mixes (*HMA_ref_*, *WMA_ref_*, *WMA_nz_*_,0.4%*S*_ and *WMA_nz_*_,1.0%*D*_), with the aim of designating the water and the frost sensitivity indices (*ITSR*).

The results of tested mechanical parameters show that the addition of natural zeolite to the tested asphalt mixtures also had a substantial impact on the indirect tensile strength of the Marshall samples. Both of the tested mixes with zeolites achieved comparable indirect tensile strengths. In the case of the dry specimens, the difference between average *ITS_dry_* values that are produced by the two zeolite-containing mixtures was only 14 kPa, whereas the results obtained in the samples subjected to a single freeze-thaw cycle, the difference was twice as high and amounted to 28.5 kPa. The strength results of the AC 16 mixes with zeolites were substantially lower than the results of the reference mixes, i.e., the *ITS_dry_* values of the zeolite mixtures were on average lower by ca. 220 kPa than *WMA_ref_* and by ca. 450 kPa than *HMA_ref_*. In terms of the *ITS_freeze-thaw_* parameter, the mixes with zeolites achieved values that were lower by ca. 250 kPa and by approx. 500 kPa in comparison to the reference mixes produced, respectively, in the WMA and HMA technology.

The descriptive statistics (Figure 10 and Figure 11), show that the WMAs containing natural zeolites exhibited the highest coefficients of variation in the tested volumetric and mechanical parameters when compared to the reference mixes, i.e., without zeolites.

**Figure 12:**
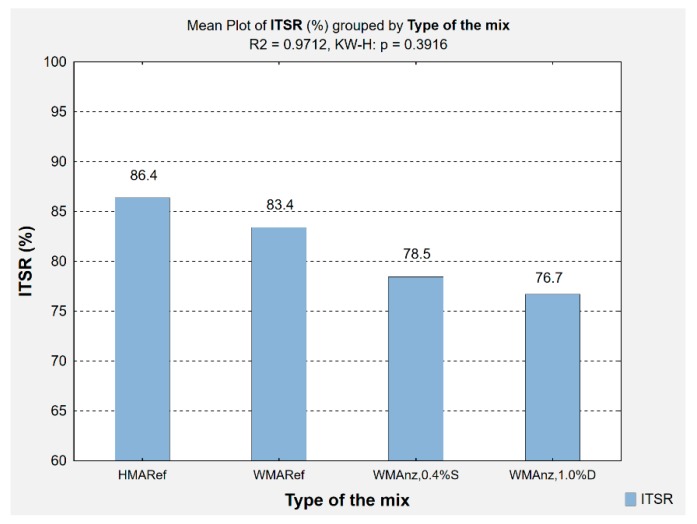
Results of water and frost susceptibility indices (*ITSR*) determination in the Marshall samples produced from the investigated AC 16.

The results of indirect tensile strength testing were used to calculate the water and frost susceptibility indices (*ITSR*), which Figure 12 presents.

The analysis of the water and frost susceptibility index after a single freeze-thaw cycle (*ITSR*) demonstrated a substantial impact of using natural zeolite, both dry and water-soaked, in the WMA mixes with foamed bitumen. In comparison to *WMA_ref_*, the WMA mix with 0.4% of water-soaked zeolite was characterised by *ITSR* that was reduced by 4.95 percent points (78.54%), whereas the mix asphalt with 1% of dry zeolite achieved an *ITSR* of 76.7%. The decreased values of the *ITSR* parameter in the samples that were produced from the asphalt mixtures with zeolites can be partially attributed to the increased air void content and greater bitumen absorptivity of zeolites than in the case of limestone powder (the mineral filler used), as confirmed by other researchers’ studies [27].

In reference to the requirements that were applicable to HMA mix asphalts, all of them met the requirements for the base course (*ITSR* > 70%) in selected CEN member states [4], whereas the reference mix asphalts (HMA and WMA) have also met the requirements for the binding layer.

#### 4.4.2. Statistical Analysis of the Test Results

A statistical analysis of the test results supplemented the estimation of the impact of using zeolites in the composition of warm mix asphalt with foamed bitumen on their physical and mechanical properties. The analyses included a deduction about the significance of differences between the average values of the measured variables (*V_a_, ITS_S_, ITS_freeze-thaw_*) in terms of mixture composition and production method (*HMA_ref_*, *WMA_ref_*, *WMA_nz_*_,0.4%*S*_ and *WMA_nz_*_,1.0%*D*_) while using the analysis of variance (one-way ANOVA). It was possible to use the *F* (Fisher-Snedecor) parametric test that enables simultaneously comparing several mean values due to the conformity of the variables’ distribution with the standard distribution and meeting the assumption of homogeneity of variances in the evaluated groups. The estimation of the significance in multiple comparisons of the differences between the tested groups was executed in a Tukey *post-hoc* test due to the fact that for all tested variables the main effects yielded statistical significance in the general *F* test. This allowed for identifying the specific mean values differing from one another.

Table 14 summarises the results of the one-way ANOVA testing for the impact of the type of mixture on the physical and mechanical test results. Table 15 presents the results of the Tukey multiple comparisons test.

The analysis of the ANOVA table and the obtained *p*-values for the *F* statistic (Table 14), with the assumed a significance level of α = 0.05, it is possible to state that the type of the asphalt mix was statistically significant in the scope of all the tested parameters. It was possible to use the multiple comparisons test to determine which mix asphalts significantly differed from one another due to the fact that all considered parameters achieved a *p*-value < 0.05.

The multiple comparisons tests demonstrated (Table 15) that the performance of the WMA mixtures containing zeolites (*WMA_nz_*_,0.4%*S*_ and *WMA_nz_*_,1.0%*D*_) can be considered to be similar to each other, i.e., the differences between the measured average values of *V_a_, ITS_d_* and *ITS_freeze = thaw_* of these mixtures were not proven to be statistically significant. Meanwhile, the *HMA_ref_* and *WMA_ref_* mixtures significantly differed from each other and the WMA zeolite mixes in the terms of both the volumetric and mechanical performance.

## 5. Conclusions

The presented research was conducted to evaluate the impact of natural zeolites on the properties of warm mix AC 16 asphalt concrete mixtures with foamed bitumen.

The first stage of the investigations included the determination of the foaming performance of the bitumen with an adhesion promoter added in 0.0–0.9% concentration. It was found that the presence of the surfactant influenced the bitumen foam half-life to a higher degree than the maximum expansion ratio. The optimum foaming content was determined at 3.0%.

The main body of conducted research concerned the evaluation of two modes of zeolite dosing to the foamed WMA mixtures: air-dry zeolites (with ca. 7.4% moisture content) added at 1.0% by wt. of the mixture and water-soaked zeolites (with 20% total moisture content) added at 0.4% by wt. of the mix. The results of these investigations allowed for the formulation of the following conclusions:

- the use of natural zeolites in the AC 16 asphalt concrete mix that was produced in the WMA technology with foamed bitumen caused a change in its volumetric and mechanical parameters when compared to the reference asphalt mixes (HMA and WMA) without zeolites;
- the addition of natural zeolites has substantially affected the compactability of WMA mixtures measured in gyratory compactor: the tests demonstrated increased air void contents by as much 1.9 to 3.9% in comparison to the reference WMA mixture;
- in the evaluation of the Marshall compacted samples, natural zeolite dosage into the asphalt concrete mix caused an increase in the air void content by ca. 2% in the case the *WMA_nz_*_,0.4%*S*_ mixture containing water-soaked zeolite in the quantity of 0.4% and by ca. 3% for the *WMA_nz_*_,1.0%*D*_ mixtures containing 1.0% of dry zeolite in relation to reference warm mix asphalt;
- the amount and mode of dosing of the natural zeolites (*WMA_nz_*_,0.4%*S*_, *WMA_nz_*_,1.0%*D*_) had no significant impact on the tested properties (*V_a_*, *ITS_dry_*, *ITS_freeze-thaw_*, *ITSR*) of Marshall compacted samples of the AC 16 foamed warm mix asphalt concrete; the difference in the *ITS_dry_* between the *WMA_nz_*_,0.4%*S*_ and *WMA_nz_*_,1.0%*D*_ asphalt mixtures amounted to 14 kPa, and for *ITS_freeze-thaw_* it amounted to 28.5 kPa, which was also a minor difference;
- the indirect tensile strengths of the samples that were produced from asphalt mixtures containing natural zeolites in comparison to the reference *WMA_ref_* mix were reduced by approx. 220 kPa in dry state (*ITS_dry_*) and by approx. 250 kPa after a freeze-thaw cycle (*ITS_freeze-thaw_*), as a result the warm mixtures containing zeolites exhibited increased susceptibility to water and frost (*ITSR*);
- based on the test results, it was inferred that the decreased mechanical parameters (*ITS_d_*, *ITS_freeze-thaw_*, *ITSR)* of the analysed WMA mixtures with foamed bitumen and zeolites were partially caused by the increased air void contents; and,
- all of the WMA mix asphalts with foamed bitumen, including those containing zeolites achieved low susceptibility to water and frost (*ITSR* > 70%) fullfilling the requirements for the base courses in the selected CEN member states [4], whereas the reference asphalt mixes (HMA*_ref_* and WMA*_ref_*) have also met the requirements for the binding layers (*ITSR* > 80%).

The study has shown that investigated WMA’s with foamed bitumen produced at ca. 120 °C did not benefit from the addition of zeolites. The warm foamed mixtures with additional doses of zeolites exhibited decreased compactability, which resulted in increased air void contents, decreased ITS strengths and moisture resistance. Based on the findings of other researchers, several explanations have been proposed for these effects. The decreased compactability could be a result of the significantly increased amount of total foaming water introduced with the zeolites, which has been previously reported to worsen the workability of WMA’s with foamed bitumen. Inferior mix performance could also stem from the increased bitumen absorptivity of the zeolite-bearing filler fraction, leaving less “free bitumen” in the mixture and, thus, rendering the designed mix composition suboptimal. It was also indicated that the simultaneous use of mechanical water foaming and introduction of zeolites could require increased mixture processing temperatures for improved performance.

Based on these premises, further works in this field are aimed at investigating the properties of the foamed WMA mixtures with different dosages of zeolites being produced at higher temperatures, as well as at broadening the scope of the tests to assess the rutting, low temperature, and stripping performance of this type of mixtures.

## Figures and Tables

**Table 1:** The framework composition of the evaluated AC 16 mixture.

AC Constituents	Material Type	
Natural Aggregates	Filler aggregate (limestone)	
	Fine natural aggregate 0/2 mm (limestone)	
	Continuously graded natural aggregate 0/4 mm (limestone)	
	Coarse aggregate 2/5 mm (quartzite)	
	Coarse aggregate 5/8 mm (quartzite)	
	Coarse aggregate 8/16 mm (limestone)	
Bituminous Binder	Paving Bitumen 35/50	Liquid (unfoamed)
		Foamed (water injection)
Additives	Adhesion Promoter (Wetfix BE)	
	Natural Zeolite	Saturated with water up to 20% (wt.)
		Dry (air-dried at room temp, RH = 60%)

**Table 2:** The calculation of total added moisture in the investigated mixtures.

Mix Type	Binder Content in the Mix	Foaming Water Content	Zeolite Water Content	Zeolite Content	Calculation of Total Added Moisture in the Mix	
*HMA_Ref_*	4.5%	-	-	-	-	
*WMA_Ref_*	4.5%	3.0%	-	-	4.5% × 3.0% = 0.135%	
*WMA_nz_*_,0.4%*S*_	4.5%	3.0%	20%	0.4%	0.135% + 0.4% × 20% = 0.135% + 0.08% =	0.215%
*WMA_nz_*_,1.0%*D*_	4.5%	3.0%	7.4%	1.0%	0.135% + 1.0% × 7.4% = 0.135% + 0.074% =	0.209%

**Table 3:** Results of sieve analysis of mineral aggregates used in AC 16 asphalt mixes.

Particle Size # (mm)	Component					
	Filler	0/2 mm	0/4 mm	2/5 mm	5/8 mm	8/16 mm
16	0	0	0	0	0	6.8
11.2	0	0	0	0	0	42.3
8	0	0	0	0	8.2	40.8
5.6	0	0	2.4	11.0	64.5	7.0
4	0	0.1	13.3	31.9	16.5	0.5
2	0	9.2	29.0	45.9	6.8	0.2
1	0	25.9	20.6	8.4	1.2	0
0.5	0	19.8	11.1	0.7	0.3	0
0.25	0.8	16.7	6.7	0.1	0.2	0
0.125	2.3	16.4	3.8	0.2	0.3	0.1
0.063	4.8	8.3	2.7	0.4	0.5	0.2
<0.063	92.1	3.5	10.4	1.4	1.5	2.0
Sum	100	100	100	100	100	100

**Table 4:** The results of EDX analysis using standardless ZAF quantification method of the natural zeolite using the Quanta FEG 250 SEM.

Elem.	wt. %	at. %	k-Ratio	Z	A	F
O K	41.18	56.15	0.1245	1.0310	0.2931	1.0005
Na K	0.07	0.06	0.0003	0.9649	0.4611	1.0059
Mg K	0.55	0.49	0.0034	0.9891	0.6209	1.0119
Al. K	7.68	6.21	0.0564	0.9600	0.7513	1.0183
Si K	42.02	32.64	0.3230	0.9880	0.7772	1.0010
K K	4.00	2.23	0.0333	0.9374	0.8848	1.0043
Ca K	2.92	1.59	0.0254	0.9596	0.9063	1.0007
Fe K	1.59	0.62	0.0137	0.8718	0.9928	1.0000

**Table 5:** Framework composition of the investigated AC 16 asphalt mixes.

Component	Percentage (wt. %)					
	Asphalt Concrete Mixture Type					
	*HMA_Ref_, WMA_Ref_*		*WMA_nz_*_,0.4%*S*_		*WMA_nz_*_,1.0%*D*_	
	mm	am	mm	am	mm	am
Filler aggregate	4	3.8	3.6	3.4	3	2.9
Fine natural aggregate 0/2 mm	15	14.3	15	14.3	15	14.3
Continuously graded natural aggregate 0/4 mm	15	14.3	15	14.3	15	14.3
Coarse aggregate 2/5 mm	12	11.5	12	11.5	12	11.5
Coarse aggregate 5/8 mm	12	11.5	12	11.5	12	11.5
Coarse aggregate 8/16 mm	42	40.1	42	40.1	42	40.1
Zeolite		-	0.4	0.4	1	0.9
Paving bitumen 35/50	-	4.5 ^(a),(b)^	-	4.5 ^(b)^	-	4.5 ^(b)^
Total		100	100	100	100	100

Surface active agent (Wetfix BE) added in the amount: ^(a)^ 0.3% (by wt.) to *HMA_Ref_* ^(b)^ 0.6% (by wt.) to *WMA_Ref_*, *WMA_nz_*_,0.4%*S*_ and *WMA_nz_*_,1.0%*D.*_

**Table 6:** The digest of sieving analysis of the designed asphalt concrete mixtures.

Feature	Asphalt Concrete Mixture Type		
	*HMA_Ref_, WMA_Ref_*	*WMA_nz_*_,0.4%*S*_	*WMA_nz_*_,1.0%*D*_
Fraction > 2 mm (%)	71.3	71.3	71.3
Fraction from 0.063 mm to 2 mm (%)	21.7	21.7	21.7
Fraction < 0.063 mm mm (%)	7.0	7.0	7.0
Density ρ_a_ (Mg/m^3^)	2.697	2.695	2.692

**Table 7:** Basic properties of 35/50 asphalt binders with variable amounts of surfactant.

Variable	WBE Percentage (%)	Descriptive Statistics					
		Valid N	Mean	Min.	Max.	Std. Dev.	CV (%)
Penetration in 25 °C (Pen_25_) (0.1 mm)	0.0	10	36.6	35.0	38.0	1.17	3.21
	0.3	10	40.8	38.3	43.6	1.59	3.90
	0.6	10	41.5	40.3	43.2	0.95	2.30
	0.9	10	42.9	40.2	45.2	1.82	4.25
Softening point (*T_R&B_*) (°C)	0.0	4	54.3	53.8	54.6	0.36	0.66
	0.3	4	53.9	53.8	54.1	0.13	0.23
	0.6	4	53.7	53.6	53.8	0.08	0.15
	0.9	4	53.4	53.3	53.6	0.13	0.24
Fraass breaking point (*T_Fraass_*) (°C)	0.0	3	−13.7	−14.0	−13.0	0.58	4.22
	0.3	3	−13.3	−15.0	−12.0	1.53	11.46
	0.6	3	−12.7	−13.0	−12.0	0.58	4.56
	0.9	3	−12.3	−13.0	−11.0	1.16	9.36

**Table 8:** Calculated penetration index (PI) and temperature plasticity range (PR) of 35/50 asphalt binders with variable amounts of surfactant.

WBE Percentage (%)	*PI*	*PR* (°C)
0.0	−0.9	68.0
0.3	−0.7	67.2
0.6	−0.7	66.4
0.9	−0.7	65.7

**Table 9:** Results of analysis of variance (one-way ANOVA) assessing the effects of WBE content on the basic properties of the tested bituminous binders.

Variable	Effect	SS	Df	MS	F	*p*-Value
Penetration in 25 °C *Pen_25_*	Intercept	52,141.68	1	52,141.68	23,150.84	<0.001
	WBE percentage	23.34	2	11.67	5.18	0.012466
	Error	60.81	27	2.25		
Softening point *T_R&B_*	Intercept	34,582.80	1	34,582.80	2,706,480	<0.001
	WBE percentage	0.50	2	0.25	20	0.000522
	Error	0.12	9	0.01		
Fraass breaking point *T_Fraass_*	Intercept	1469.444	1	1469.444	1102.083	<0.001
	WBE percentage	1.556	2	0.778	0.583	0.586816
	Error	8.000	6	1.333		

Df—degrees of freedom, SS—Sum of Squares, MS—Mean squares.

**Table 10:** Foaming performance of the reference 35/50 penetration bitumen.

FWC	Valid N	ERm (−)					HL (s)				
		Mean	Min.	Max.	Std. Dev.	CV (%)	Mean	Min.	Max.	Std. Dev.	CV (%)
1.5%	4	7.3	7.0	8.0	0.50	6.90	38.3	37.0	40.0	1.50	3.92
2.0%	4	9.5	9.0	10.0	0.58	6.08	32.8	31.0	34.0	1.26	3.84
2.5%	4	11.8	11.0	13.0	0.96	8.15	27.5	26.0	29.0	1.29	4.69
3.0%	4	14.3	13.0	16.0	1.26	8.83	24.5	23.0	26.0	1.29	5.27
3.5%	4	16.3	15.0	18.0	1.26	7.74	20.3	19.0	21.0	0.96	4.73

**Table 11:** Statistical analysis of the model predicting ERm of 35/50 foamed bitumen based on the foaming water content, WBE content, and their interaction.

Variable	Estimate	Std. Error	T Value	Pr(>|t|)
Intercept	−2.78393	1.82614	−1.5245	0.13042
WBE	3.32847	2.89098	1.1513	0.25222
FWC	5.41563	0.70269	7.7069	0.00000
WBE * FWC	−0.72934	1.11274	−0.6554	0.51364
Regression Summary: *ERm* (35/50 + WBE) Multiple R^2^: 0.8333, Adjusted R^2^: 0.8285 *F*-statistic: 173.3 on 3 and 104 DF, *p*-value: < 2.2 × 10^−16^				

**Table 12:** Statistical analysis of the model predicting HL of 35/50 foamed bitumen based on the foaming water content, WBE content and their interaction.

Variable	Estimate	Std. Error	T Value	Pr (>|t|)
Intercept	31.7447	4.3541	7.2907	0.00000
WBE	22.1424	6.8931	3.2123	0.00175
FWC	−5.8413	1.6755	−3.4864	0.00071
WBE*FWC	−3.8837	2.6531	−1.4638	0.14626
Regression Summary: *HL* (35/50 + WBE) Multiple R^2^: 0.7364, Adjusted R^2^: 0.7288 *F*-statistic: 96.86 on 3 and 104 DF, *p*-value: < 2.2 × 10^−16^				

**Table 13:** Results of the compactability analysis of the AC 16 mixes compacted in the gyratory compactor; mean values of the samples’ air voids content *V_a_*.

No. of Gyrations	Air Void Content *V_a_* (%)			
	*HMA_ref_*	*WMA_ref_*	*WMA_nz_*_,0.4%*S*_	*WMA_nz_*_,1.0%*D*_
10	16.23	16.71	18.77	20.06
20	13.69	14.22	16.26	17.56
40	11.02	11.58	13.55	14.72
80	8.44	8.85	10.99	12.04
128	6.76	7.16	9.33	10.36
200	5.33	5.67	7.88	8.74
320	4.09	4.41	6.54	7.35
512	3.12	3.48	5.40	5.99

**Table 14:** The results of one-way ANOVA investigating significance of the type of mixture (*HMA_ref_*, *WMA_ref_*, *WMA_nz_*_,0.4%*S*_ and *WMA_nz_*_,1.0%*D*_) in relation to the dependent variables: *V_a_, ITS_dry_, ITS_freeze-thaw_*.

Dependent Variable	Effect	df	SS	MS	F	*p*-Value
*V_a_*	Intercept	1	1328.256	1328.256	7246.132	<0.001
	Type of mixture	3	30.395	10.132	55.272	<0.001
	Error	36	6.599	0.183	−	−
*ITS_dry_*	Intercept	1	43,812,546	43,812,546	3923.329	<0.001
	Type of mixture	3	1,121,516	373,839	33.477	<0.001
	Error	28	312,681	11,167	−	−
*ITS_freeze-thaw_*	Intercept	1	28,493,702	28,493,702	2730.283	<0.001
	Type of mixture	3	1,463,382	487,794	46.741	<0.001
	Error	28	292,213	10,436	−	−

df—degrees of freedom, SS—Sum of Squares, MS—Mean squares.

**Table 15:** Results of post-hoc Tukey multiple comparison test.

Tukey HSD Test, Variable ***V_a_*** (%) Approximate Probabilities for Post Hoc Tests Error: Between MS = 0.18331; df = 36					
**Type of Mix**		**{1} 4,41**	**{2} 5,61**	**{3} 6,35**	**{4} 6,68**
*HMA_ref_*	{1}	-	0.000159	0.000159	0.000159
*WMA_ref_*	{2}	0.000159	-	0.002522	0.000174
*WMA_nz_*_,0.4%*S*_	{3}	0.000159	0.002522	-	0.326804
*WMA_nz_*_,1.0%*D*_	{4}	0.000159	0.000174	0.326804	-
Tukey HSD Test; Variable ***ITS_dry_*** (kPa) Approximate Probabilities for Post Hoc Tests Error: Between MS = 11167; df = 28					
**Type of Mix**		**{1} 1452.7**	**{2} 1225.6**	**{3} 1008.2**	**{4} 993.94**
*HMA_ref_*	{1}	-	0.001146	0.000164	0.000164
*WMA_ref_*	{2}	0.001146	-	0.001767	0.000936
*WMA_nz_*_,0.4%*S*_	{3}	0.000164	0.001767	-	0.993100
*WMA_nz_*_,1.0%*D*_	{4}	0.000164	0.000936	0.993100	-
Tukey HSD Test; Variable ***ITS_freeze-thaw_*** (kPa) Approximate Probabilities for Post Hoc Tests Error: Between MS = 10436; df = 28					
**Type of Mix**		**{1} 1255.2**	**{2} 1022.2**	**{3} 790.90**	**{4} 762.39**
*HMA_ref_*	{1}	-	0.000640	0.000164	0.000164
*WMA_ref_*	{2}	0.000640	-	0.000684	0.000168
*WMA_nz_*_,0.4%*S*_	{3}	0.000164	0.000684	-	0.364158
*WMA_nz_*_,1.0%*D*_	{4}	0.000164	0.000168	0.364158	-
